# Endometriosis in a postmenopausal woman without previous hormonal therapy: a case report

**DOI:** 10.1186/1752-1947-3-135

**Published:** 2009-11-18

**Authors:** Manuel García Manero, Pedro Royo, Begoña Olartecoechea, Juan Luis Alcázar

**Affiliations:** 1 Department of Obstetrics and Gynecology, Clínica Universitaria de Navarra, Avenida Pío XII, 36, 31008 Pamplona, Spain; 2 Department of Obstetrics and Gynecology, Hospital San Jorge de Huesca, Avenida Martínez de Velasco, 22004 Huesca, Spain

## Abstract

**Introduction:**

The prevalence of pelvic endometriosis is high, affecting approximately 6% to 10% of women of reproductive age. Although endometriosis has been associated with the occurrence of menstrual cycles, it can affect between 2% to 5% of postmenopausal women.

**Case presentation:**

We present a case of ovarian endometriosis in a 62-year-old Spanish Caucasian woman with no previous use of hormonal therapy and no history of endometriosis or infertility.

**Conclusion:**

Although the reported situation is rare, it is important to be aware of endometriosis after the menopause: post-menopausal endometriosis confers a risk of recurrence and malignant transformation.

## Introduction

Endometriosis is a common, benign, estrogen-dependent, chronic gynecological disorder commonly associated with pelvic pain and infertility. The prevalence of pelvic endometriosis is high, affecting approximately 6% to 10% of women of reproductive age [1]. Although endometriosis has been associated with the occurrence of menstrual cycles, it can affect between 2% to 5% of postmenopausal women [2], and generally occurs as a side effect of hormone use [3,4]. In these cases, a differential diagnosis to exclude malignancies is critical. However, endometriosis can also occur in postmenopausal women not receiving exogenous hormones, indicating the complex pathogenesis of endometriosis. In clinical practice, the discrimination between endometriosis and cancer is further complicated by the fact that some of the risk factors for endometriosis and ovarian malignancy are similar: a low rate of parity, infertility, late childbearing age, and a short duration of oral contraceptive use [5]. Although there are some reports of successful results with treatments such as aromatase inhibitors [6], we think that surgery should be the first step in the management of postmenopausal ovarian endometriosis.

We present a case of ovarian endometriosis in a postmenopausal woman with no previous hormonal therapy (HT) use and no history of endometriosis or infertility.

## Case presentation

**Figure 1:**
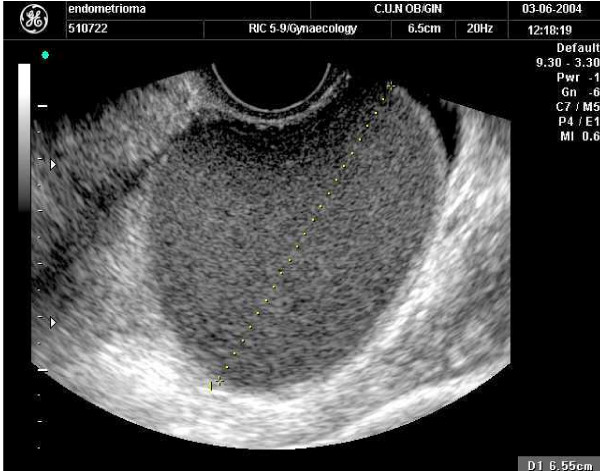
**Ultrasound imaging of the ovarian cystic lesion**.

A 62-year-old, non-obese, Spanish Caucasian woman presented with acyclic pelvic pain. The patient's menarche occurred when she was 13 years old and her menopause at 47. She denied current or previous use of HT or a prior history of pelvic pain or dysmenorrhoea. She had no familial or personal history of endometriosis. A physical examination revealed a regular increased sized left adnexa as a unique pathologic feature. A pelvic ultrasound scan revealed a left ovarian homogeneous cystic mass of approximately 4.4 × 2.7 × 2.7 cm in size (Figure 1). The Doppler blood flow study suggested a benign ovarian mass. The cancer antigen serum markers (cancer antigen 125, alpha-fetoprotein, squamous cell carcinoma, carcinoembryonic antigen) were negative. The data suggested a provisional diagnosis of left ovarian endometrioma.

**Figure 2:**
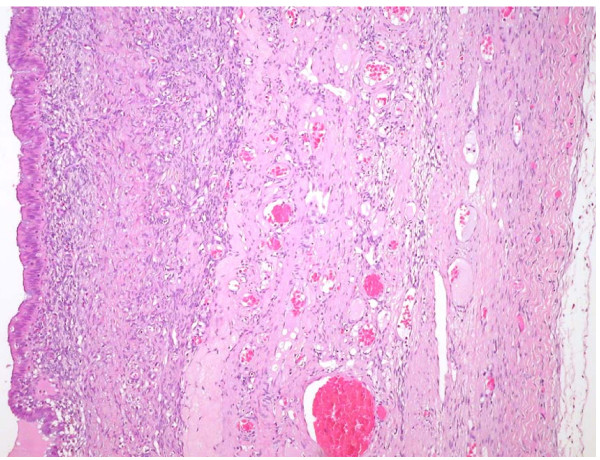
**Microscopic aspect of the ovarian lesion**.

Laparoscopy revealed a cystic left adnexal mass; no adhesions or other pelvic endometriotic lesions were observed. She was submitted to a bilateral laparoscopic salpingoophorectomy, and subsequent histological analysis confirmed an ovarian endometriotic cyst (Figure 2).

## Discussion

Postmenopausal endometriosis was first reported in 1950. Although a rare disease, it should be considered in postmenopausal and women who have undergone hysterectomy with classical symptoms of endometriosis, mostly pain.

In the presence of adnexal masses in postmenopausal women, the gynecologist must always consider the possibility of a malignant ovarian tumor. In spite of being an uncommon disease after menopause, endometriosis, which is known to be estrogen-dependent, is been included in the list of possible differential diagnoses when dealing with postmenopausal women. In these cases, the theoretical celomic metaplasia etiopathogenic mechanism [7,8] could explain the occurrence of postmenopausal ovarian endometriotic lesions. Another possible explanation is endometrial stem cells from vascular endometrial cell transportation, which occurs primarily when endometriotic lesions appear in areas that do not have contact with menstrual retrograde flow [9,10].

These investigations suggest that some interleukins (interleukin (IL)1, IL2, IL6, IL8, IL10) and other inflammatory mediators (tumor necrosis factor alfa, interferon gamma, monocyte chemotactic protein-1) could play a main role in the endometriosis pathophysiology, allowing ectopic endometrial cells to implant and grow or triggering a celomic metaplasia etiopathogenic mechanism. We postulate that some postmenopausal women could have a relative immunosuppression status that allows the lesions to establish and progress [11].

Although the condition is rare, it is important to be aware of endometriosis after menopause. Postmenopausal endometriosis confers a risk of recurrence and malignant transformation. Some endometriosis lesions may predispose to clear cell and endometrioid ovarian cancers. Ovarian endometriomas that are 9 cm or greater in diameter are a strong predictor for development of ovarian cancer in postmenopausal women of 45 years of age or older [12].

Although conclusive evidence is lacking, the risk of malignant transformation appears to be lower with combined HT compared with estrogen-only therapy. Thus, hormone replacement therapy should generally be reserved for patients with severe climacteric complaints, and if indicated, combined therapy should be used [13].

## Conclusion

Although the reported situation is rare, it is important to be aware of endometriosis after the menopause: post-menopausal endometriosis confers a risk of recurrence and malignant transformation

## Abbreviations

HT: hormone therapy.

## Consent

Written informed consent was obtained from the patient for publication of this case report and any accompanying images. A copy of the written consent is available for review by the Editor-in-Chief of this journal.

## Competing interests

The authors declare that they have no competing interests.

## Authors' contributions

MM managed the case and wrote the report introduction, description and discussion. PR and BO reviewed the literature related and were responsible for the final manuscript form. JA, as a relevant specialist in obstetric and gynecology, revised and corrected all relevant areas of the text. All authors read and approved the final manuscript.
